# Diversity in Defining End of Life Care: An Obstacle or the Way Forward?

**DOI:** 10.1371/journal.pone.0068002

**Published:** 2013-07-03

**Authors:** Marjolein Gysels, Natalie Evans, Arantza Meñaca, Irene J. Higginson, Richard Harding, Robert Pool, on behalf of Project PRISMA

**Affiliations:** 1 Centre for Social Science and Global Health, University of Amsterdam, Amsterdam, The Netherlands; 2 Department of Palliative Care, Policy and Rehabilitation, King’s College London, London, United Kingdom; 3 Barcelona Centre for International Health Research, Universitat de Barcelona, Barcelona, Spain; 4 Department of Public and Occupational Health, Emgo Institute for Health and Care Research, Expertise Center for Palliative Care, VU University Medical Center, Amsterdam, The Netherlands; Edinburgh University, United Kingdom

## Abstract

**Aim:**

The terms used to describe care at the end of life (EoL), and its definitions, have evolved over time and reflect the changes in meaning the concept has undergone as the field develops. We explore the remit of EoL care as defined by experts in EoL care, from across Europe and beyond, to understand its current usage and meanings.

**Method:**

A qualitative survey attached to a call for expertise on cultural issues in EoL care was sent to experts in the field identified through the literature, European EoL care associations, and conferences targeted at EoL care professionals. Respondents were asked to identify further contacts for snowball recruitment.The responses were analysed using content and discourse analysis.

**Results:**

Responses were received from 167 individuals (33% response rate), mainly from academics (39%) and clinical practitioners working in an academic context (23%) from 19 countries in Europe and beyond. 29% of respondents said explicitly that there was no agreed definition of EoL care in practice and only 14% offered a standard definition (WHO, or local institution). 2% said that the concept of EoL care was not used in their country, and 5% said that there was opposition to the concept for religious or cultural reasons. Two approaches were identified to arrive at an understanding of EoL care: exclusively by drawing boundaries through setting time frames, and inclusively by approaching its scope in an integrative way. This led to reflections about terminology and whether defining EoL care is desirable.

**Conclusion:**

The global expansion of EoL care contributes to the variety of interpretations of what it means. This complicates the endeavour of defining the field. However, when diversity is taken seriously it can open up new perspectives to underpin the ethical framework of EoL care.

## Introduction

The terminology used to describe care for people with a life-limiting and progressive illness has changed over time from care of the dying to terminal care, hospice care, palliative care, and in some contexts to supportive care [1]. These terms reflect the historical development of care at the end of life (EoL) and the changing meanings the concept underwent as the expertise in this area developed. The term ‘hospice care’ developed from Cicely Saunders’ ideas about total care, which she thought needed to be provided in institutions separate from mainstream hospitals, which were primarily directed towards curative interventions [2]. Although ‘palliative care’ dates back to the seventeenth century, the term entered common usage when it was used to refer to hospice-type care provided in other settings such as hospitals and the community [3], [4].

The terms used were also determined by their acceptability to patients and families. ‘Hospice care’ connotes death and dying and ‘palliative care’ has also become associated with death [5]. Instead, the term ‘supportive care’ is often preferred when referring patients to specialist palliative care services [6], [7]. This is because, although encompassing palliative care, it also includes the minimisation of treatment side-effects and does not exclude the possibility of survival [8]. Patients view the term more positively as it implies active intervention and therefore leaves space for hope.

Definitions for care at the EoL have broadened considerably over time [1], both in regard to the point in the illness trajectory when EoL care should be introduced, (from the last phase of illness to diagnosis) and in terms of the clinical condition on which it focuses (from cancer to all chronic or life-threatening illnesses). ‘Palliative care’ has also become increasingly secularized and universal bioethical principles have replaced its original religious values of ‘sanctity of life’ or ‘love’.

Currently a multiplicity of terms and definitions to refer to the concept of ‘EoL care’ exist alongside each other [9]. Several papers have pointed out that the diversity in terminology and definitions is problematic as it hampers the development of this field academically, clinically and adminstratively [10]. To come to grips with the various definitions, analyses focus on specific terms in the literature and call for standardisation of terminology and definitions [11].

Here we take a novel approach towards understanding definitions of care at the EoL, which traditionally focused on particular terms as they were defined in the literature, often in a specific specialisation, such as palliative care or oncology. In this paper, we explore the remit of care at the EoL as it is defined in different countries based on the reflections of experts from across Europe and beyond. Such a broad approach allows us to escape the predetermined categories from which definitions are usually formulated in the literature, and to explore not only the areas of agreement but also of diversity. In this paper, we will use the term ‘EoL care’ to refer to the broad descriptive meaning of this type of care, separate from the diverse associations it has acquired historically.

## Methods

This research was conducted in the context of the project PRISMA [12], which enquired about ideas and practices of care at the EoL in diverse societies.

### Design

A qualitative survey, as part of a call for expertise on cultural issues in EoL care, was sent to European experts in EoL care. The purpose of the call for expertise was to build a network of experts on cultural issues in EoL care. The qualitative survey was appended to the call for expertise and consisted of five open questions exploring the areas of agreement and difference in ideas and practices of EoL care across countries.

### Content

The survey consisted of five questions: 1. How broadly is EoL care defined in your country? 2. What role does culture play in EoL care in your country? 3. To what extent is EoL care taken into account in policy and practice in your country? 4. What is the most important issue relating to culture and EoL care that you think needs to be addressed in your country? 5. In what way is the approach to dying in your country different from other European countries? They were translated into Spanish, Italian, Portuguese, German and Dutch by native speakers. This paper focuses only on responses to the first question on definitions.

### Recruitment

In order to contact experts in culture and EoL care in Europe, the survey was first sent by e-mail to experts in culture and EoL care known to the research team, and to those identified from reviews of the literature [13], [14], [15], [16], [17], [18], [19]. It was also sent to all European palliative care associations and to conferences and workshops targeted at palliative care professionals covering issues on culture at the EoL. The call for expertise was published in newsletters of Hospice UK Online, Worldwide Hopsice and Palliative Care Online, UK Palliative Care Research Society, and European Network on Intercultural Elderly Care. The call incorporated a snowball sampling approach by asking participants to refer to other experts. It was conducted over the period of a year in 2009–2010.

### Analysis

The analysis consisted of the following steps:

1. The answers to the survey questions were imported into NVivo (computer software for the management and analysis of textual data). Data on country and occupation were entered in an Excel spreadsheet.
2. Answers were read and coded by two members of the team based initially on a preliminary coding scheme derived from the central topics on which the questions focused (e.g. types of definitions, criteria). This coding was then compared and discussed between the coders and more widely in the team in order to resolve any differences. Subsequent readings led to the development of additional codes. The main coding scheme for the question on definitions was:

- Types
  1. Standard
  2. Unclear
  3. Lack of concept
- Criteria (that constitute definitions for delineation)
  1. Timing
    1. Determined by delineation (policy/, practice)
    2. Narrow versus broad
    3. Transitions (problem of delineating, fixing time by determining moment of death)
    1. Personhood
    1. Consciousness
    2. Social death
  2. Medical EoL decisions (terminal care)
- In/exclusive
  1. Type of illness, skills
- Consequences (of focus or absence of definitions)
  1. For defining
  2. Against defining
- Terms
  1. End of life care
  2. Culture-specific terms
- Values
  1. Includes euthanasia
  2. Quality of life

Because the development of the coding scheme was iterative, the final set of codes also represents the main themes that emerged from the answers.

1. Sections of text coded according to the main topics above were then extracted and where relevant frequencies were noted. Comparisons were made within the coded sections between responses from different countries, and the centrality, importance and meaning of the various coded topics in the answers were assessed.

### Ethics Approval

Ethics approval was obtained for this work package of the PRISMA programme from the ethics committee of the Fundacio Clinic in Barcelona (Ref. nr. 2009/4778).

## Results

A total of 511 questionnaires were sent to people identified as experts, of which 167 questionnaires (33%) were returned. Of these 161 had answers to the question on definitions.

Almost half (49%) of all respondents had been recommended by other experts (‘snowballing’), just under a third had been identified from publications (32%), 14% of respondents had responded to the call for expertise whereas 5% were identified during conferences on EoL care. The number of people contacted via each method also follows this descending order (Table S1).

The average response rate for the whole questionnaire was 33%. The largest number of people contacted came from the UK (68), Spain (65) and Germany (64); the highest percentage of total responses came from the UK (20%), Spain (13%) and the Netherlands (12%) (Table S2). Responses were seven lines long on average, the median was four (range 1–116 lines), and the mode was two lines (Table S3).

The majority of respondents were academics (39%), followed by academics who were also clinical practitioners (23%), and clinical practitioners (17%) (Table S4).

### The EoL in Practice

Twenty nine percent of experts stated explicitly that there was no agreed definition of EoL care in their country.

Fourteen percent of respondents said they based their understanding of the EoL on standard definitions of care, mostly the WHO definition of palliative care. In the UK, Spain and Belgium, respondents also used definitions from national associations or policy.

Criteria for deciding when EoL care is necessary depend in practice on a number of factors:

*Laws on EoL care and euthanasia, eligibility criteria in insurance policies, scientists, care providers. Variations across disciplines, gaps between policy and practice and preferences depending on providerś perspectives… (Academic, Belgium, Be5).*

Two percent of respondents said that the concept of EoL did not exist, or was not used or discussed in relation to care in their country (India (1); the Netherlands (2); and Uganda (1)):

*…culturally speaking, the notion of “end-of-life” does not resonate well with the deeply rooted belief in “life after death” held by many Indians.* (Academic, India, In1).

Five percent of respondents talked about opposition to the concept (as opposed to curative care) (Argentina (1), Israel (1), Spain (3), Uganda (3)):

*End of life remains a “taboo” subject in our country. The reason might be because of the value of life according to the Jewish religion.* (Politician, Israel, Is1).

Respondents provided their own perspectives on the variations in understanding EoL care and what it should comprise. There were two main ways in which experts defined EoL care: exclusively, by determining its boundaries, and inclusively, by considering its scope in terms of the range of skills it requires due to the complexities involved. This led to reflections on whether defining EoL care is desirable and on the terminology.

### Determining Boundaries by Setting Time Frames in Definitions

Respondents said that the time attributed to the EoL differed across settings and sectors of care, depending on the perceptions or motivations of the parties involved. Time frames are set much broader and less specifically in policy than in practice. In policy, the EoL is contemplated in ideal terms, a scenario responding to the goal of EoL care to allow for the best possible care for the patient.

*Among policy makers and experts, the importance of needs assessment and palliative care provision for those who have no curative options but have reasonable life expectancy (e.g. a year) is evident. However, the implicit ‘culture’ dictates that end-of-life care is provided close to the actual end of life* (Academic, the Netherlands, Ne7).

Institutions however, work according to regulatory limits regarding the provision of EoL care, which are often set by financial and practical considerations.

*Home care organisations set a three month prognosis limit, but a terminal phase does not let itself be marked off in a pre-determined period* (Practitioner, the Netherlands, Ne14).

Respondents mentioned that the concept of EoL care had broadened over time, and that care takes over when cure is no longer possible. Others considered the introduction of care with a palliative purpose suitable from the point of diagnosis. However, there was a split between those who saw the EoL beginning when cure is no longer an option and those who delineate the EoL as that short period before death. For respondents from Spain, who also suggested varying timeframes, the EoL was often limited to a short time before death.

*There is a deep-rooted culture of ‘terminality’ […] (while) in England where they are working seriously with the concept of end of life (care) and taking a broad approach* (Physician, Spain, Sp 12).

The EoL is often determined based on projections associated with certain conditions. However uncertain, these prognoses are of a clinical nature, interpreted through the symptoms the patient presents. Some respondents said that chronic illness and old age are included in EoL care but these make set time frames even more problematic. Boundaries shift with the inclusion of illnesses such as dementia.

*It is the time between the threat of death and death itself. In a sudden death this can be minutes, in an unforeseen death due to acute deterioration hours or days, in a cancer trajectory weeks or months, in a chronic illness (re-occuring cancer) or degenerative illness this can be years (in the case of dementia the end of life is not so much perceived by the patient but becomes an issue for the family)* (Physician, Spain, Sp 15).

In the Netherlands, three of the ten responses referred to a narrow interpretation of EoL care, focusing on the last period before death. This interpretation is based on the Dutch law regarding medical interventions at the EoL, which concern the larger category of decisions at the EoL including euthanasia, physician assisted dying, palliative sedation, withdrawing and withholding nutrition, administration of potentially life-shortening pain relief, artificial ventilation etc [20]. This categorisation gives evidence of a history of public and professional debate about the ethical use of medical interventions when decisions become a matter of life and death [20], [21].

*The interaction between these disciplines (legal and medical) made physician-assisted dying (PAD) a possibility under well-defined and exceptional circumstances. This interaction of the courts and the medical association have helped to develop criteria for more choice at the end of life…* (Physician, The Netherlands, Ne5).

Those respondents who focused on the period just before death said that this experience with the management of EoL is a source of expertise and inspiration for liberalisation in other countries. Other Dutch respondents who embraced the palliative care view recommended the extension of the EoL forward in time, to include all aspects of the EoL and not only referring to medical interventions. However, they stressed the importance of focusing not only on a good life or quality of life, but also on dying and a good death.

*[EoL care] should be used in a more extended way to include all aspects of the end of life and not only referring to medical interventions. For example, we must be (again) in search of a good death and dying (a new art of dying) in a broad sense and not only focus on the more active decisions* (Academic, The Netherlands, Ne6).

### The Scope of EoL Care: Thinking in an Integrative Way

In addition to drawing boundaries, respondents also used a more integrative approach to delineating EoL care, by conceiving it as care that should be comprehensive due to the complexities involved. Respondents named conditions, or they characterized symptom patterns that needed EoL care:

*[EoL care] usually applies to conditions such as all types of advanced cancer, end stage heart disease, COPD, renal failure, MND, MS, dementia, AIDS etc and now includes any patients that have complex or uncontrolled psychological or physical symptom problems* (Practitioner, UK, Un20).

Respondents said that the family needed to be included in definitions, as requiring attention at the end of the patient’s life and into bereavement. Families were mentioned as decision makers, and were assigned an especially important role in paediatric palliative care and dementia.

Also the range of skills were referred to, which are needed in the provision of EoL care.

*It includes management of pain and other symptoms and provision of psychological, social, spiritual and practical support.* (Academic, UK, Un27).

One respondent wrote that biological and psychosocial influences shape the concept and determine the boundaries of care, rather than the definition providing guidance to practice.

Even when understanding EoL care in its narrowest sense, as care for the dying, some respondents stated that care transcends the point of biological death. The example of people in coma was given to show the uncertainty around the limit to life. Some brought the notion of consciousness into the debate, frequently relating levels of conciousness and quality of life to criteria for assisted dying. At the same time, this discussion led to considerations of different forms of death, such as social and psychological death. The latter was defined as the moment that a person realizes: ‘I am going to die’. The extent of suffering this brings is dependent on the various biological, social, cultural circumstances that frame the experience, and how the person perceives this.

*‘Technically, life ends when brain activity stops, but the human being as a person can disappear already long before.’* (Physician, Spain, Sp3).

Social death is considered to occur when a patient is left to die alone by their social environment, or treated as if already dead. Withholding of support by the social environment then leads to an unprepared process of dying.

*If the patient is abandoned, she could become socially dead much sooner than the subsequent phases of [physical] death. At times, (feelings of) abandonment and loneliness become so great and so unbearable that death itself comes as a relief. Amongst cancer or AIDS patients, as if they [had an] illness that was shameful in a way, it isńt rare for families to hide them at home or in the hospital. Their friends stop visiting, leaving them isolated. The social trend involves the social marginalization of a person before he or she falls ill and dies. When there is a social and personal rejection of death, there are sudden deaths for which there has been no preparation or attempts to come to terms with it.* (Academic, Spain, Sp 7).

### To Define or not to Define

Some reflections regarding the boundaries of EoL care (when it should be introduced, and to whom it should be applied, and when it has reached its limits) also included views on the nature of these boundaries and, inevitably, EoL care’s relation to medicine. They specified that boundaries should be soft, and gradual to realise the desired outcomes.

*‘Lately it (EoL care) is seen as a more gradual process where palliative care takes over from curative care in a gradual way.’* (Researcher, the Netherlands, Ne2).

Others reflected on the impossibility of fitting palliative care into a time frame. Also, when transitions were not well coordinated and smooth, this could have negative consequences.

*‘[…] palliative care and information is given only once medical treatments are given up. Overall, there is not so much cross fertilisation/communication/continuity between those two fields of activity, causing EoL care often to be considered as an “extra”. This also has a big influence on the stigmatisation of patients, who are “given up” (in a medical sense) or labelled “destitute” when they get in touch with end of life care.’* (Academic, Belgium, Be10).

The complexities that are involved in attempts to grasp the remit of EoL care and the ambiguities surrounding its borders led to some respondents doubting whether a definition of EoL care is an achievable or worthwhile goal:

*A definition would be an obstacle to the creativity and sensitivity of caregivers and distract them from their attention (to the person who needs care).* (Practitioner, Germany, Ge4 ).

However some respondents stressed that a definition of the field was very important and that it could have far-reaching consequences for its understanding and future development.

*But, there is a lack of agreement generally about what the term means, which means it is difficult to operationalise for providers and commissioners of care* (Academic, UK, Un27).

### Terminology

A variety of terms were used when referring to EoL care: ‘EoL care’, ‘palliative care’ ‘terminal care’, ‘supportive care’, ‘advanced care’, ‘advanced care planning’, ‘shared care’. These reflect a diversity of opinions of what care at the EoL should do. ‘Palliative’, ‘EoL’ and ‘terminal care’ are often also used interchangeably, not taking the differences these imply into account.

Respondents pointed out differences in meaning between ‘EoL care’ and ‘palliative care’:

*Palliative care and end of life care are both used to define patients ‘at the end of their lives’, however end of life care is used more in research settings and ‘palliative care’ seems associated with cancer as underlying disease* (Academic, the Netherlands, Ne3).

‘EoL care’ was often used as the term to refer to the comprehensive model of care which ‘palliative care’ traditionally promotes. It was then used in the broadest sense possible, including related community care and experiences. Palliative care is seen here as only one component.

‘…EoL care refers to all forms of care that relate to death, dying and loss, these are palliative care, aged care, intensive care, accident and emergency care, disaster management, coronial work, bereavement care, and funeral work.’ *(Academic, UK, Un33).*

However, its use was not consistent and in some instances the term also signaled the very last stage of life. It was mentioned that at the same time it is common to see the term, both in a policy and academic context, being used as ‘*a euphemism for hospice and palliative care’* (Academic, UK, Un33).

We encountered some country-specific concepts. In the context of the UK, the differentiation between specialist and generalist palliative care was used, which reflects its historical development primarily as specialized palliative care services. More recently, it was recognized that health and social care professionals other than specialist caregivers are involved in providing EoL care, and also in the context of the shift in policy that seeks to increase care in the community [22], generalist care at the EoL has become a major focus of health policy in the UK [23]. The concept is often defined in negative terms, as the type of care not provided by specialist interdisciplinary teams, for example including specifically trained consultants in palliative medicine, nurse specialists, specialist social workers and experts in psychological care [24].

*By ‘generalists’ we mean practitioners whose working remit is not exclusively concerned with specialist palliative care. This includes those working within primary, secondary, tertiary care, social care and the voluntary sector, and includes many who are specialists in their own sphere of expertise. Where working remits also include care of those with chronic, acute or minor illnesses, these are defined as ‘generalist’.* (Academic, UK, Un13).

In Belgium ‘integral palliative care’ was used, a term coined by the Flemish Palliative Care Federation to capture the approach to care at the EoL as it developed in the Belgian context. In ‘integral palliative care’, euthanasia and palliative care are neither alternatives nor antagonistic, but the palliative care framework embraces the option for euthanasia [25].

## Discussion

The responses to the questionnaire show that there is no agreed definition of EoL care in practice. The WHO definition [26], [27] of ‘palliative care’ is used internationally as the master definition, describing its overall approach and goals. Other definitions in particular countries are drawn upon as they are closer to the policies in the context of which stakeholders are working. Most respondents provided their own tentative, informal definitions of EoL care each emphasizing different elements they considered most important to specify its meaning.

Common elements in the definitions concern the goals to optimize quality of life and the prevention and relief of suffering through a holistic approach which requires multi- and interdisciplinary attention to those who are affected by life-threatening, advanced, and progressive illness. However, even at this level there is a lot of ambiguity and each element is open to interpretation. These ambiguities become especially apparent when attempting to delineate this type of care: at what point does care need to take over from cure, who is ill enough to be eligible for EoL care, what are the limits to carers’ responsibilities? In response, specific time frames for EoL care are drawn, based on clinical prognosis, but varying depending on the condition [28]. These are mostly uncertain and lacking evidence of reliable prognostication [23], [29], which was reflected in the diversity of participants responses, with time frames ranging from years to the final minutes before death. In practice, time limits for EoL care are usually drawn by regulatory motivations of care-providing institutions, and these tend to be a barrier to good care [30].

The other approach respondents applied to describing EoL care was integrative instead of divisive. In this way, by thinking inclusively, it becomes possible to break through conventional categories, which are generally disease-specific, and cure versus care oriented. Such a broad view is consistent with the current broadening of the remit of EoL care to non-cancer conditions where prognoses are even more difficult to establish [23]. Different transitions become apparent [31] and these need to be well coordinated, such as the transition between the curative and the palliative care settings. This also leads to a broadening on other levels such as the expertise required to work with the complexities this field presents [32].

A second reason for a broad approach to EoL care is that it can facilitate understanding of the variety of meanings attached to EoL care. Previous studies showed how differences in terms and definitions reflected the historical development of ideas about care at the EoL. This study shows how understandings differ along geographical, institutional, professional, and personal lines. From among those, we identified cultural patterns and some culture-specific definitions. This is the result of the process of modification palliative care underwent when it spread over Europe and other regions [33]. A minority of respondents mentioned the lack of or opposition to the concept. Palliative care did not maintain its original position as a separate approach to care for the incurably ill but was most often integrated into the existing health care structures of particular countries [33]. Together with pre-existing care traditions, this brought about changes in the original concept of ‘palliative care’. One example is the concept of ‘integral palliative care’ in Belgium, which is radically different from the original concept of palliative care as it embraces the option for euthanasia [25].

A broad approach to EoL care is also the most suitable response to the evolving nature of the concept. Recently, the term ‘EoL care’ has become more widely used to refer to this extended approach, encompassing palliative and supportive care and care for non-cancer patients, especially in policy [34]. Whether the term ‘EoL care’ is the most appropriate to refer to this broad, integrated approach remains to be evaluated. The disadvantage of the term ‘EoL care’ is its more narrow use to indicate the stage close to death, which coexists with this recent broader interpretation, and leads to confusion. Also, the words ‘end of life’ are problematic when used in practice in contexts where care is provided to patients and families, especially in conditions that have a chronic character.

Such an overarching notion needs to take acount of the different interpretations of EoL care such as those found in this study. The most recent WHO definition is directed to promoting EoL care on a global level [26] and therefore needs to be sensitive to cultural variations in understanding of EoL care in a diversity of settings. This can contribute to an awareness about different interpretations of EoL care and add to the inclusion of those who need this care. This applies to national differences in ideas and practices of EoL care, embedded in health care systems and supported by laws. But it is also important to understand how people from varied ethnic backgrounds relate to definitions and how these affect them. As yet there is little research on whether terms and definitions cover the concerns of minority ethnic groups [14], [15].

### Defining EoL Care is an Ethical Issue

Defining EoL care is extremely difficult because it is more than a technical delineation of a field of expertise. Determining what good care is –and here what good EoL care is, is a moral undertaking [35], [36]. EoL care has its own specific ethical values which are different from medical ethics [37]. However, its ethical basis is underexplored [38] and it is lacking the language to capture this. Scientific, objective terms, which were readily available to EoL care as it originally developed from and is still part of medicine, have filled this gap. The WHO definition, for example, uses terms such as ‘identification’, ‘assessment’ and ‘treatment’ to specify the means of realising good care, which makes EoL care primarily a medical specialisation. These terms are insufficient to grasp the specificity of EoL care and leave its moral basis unexplored.

The many different understandings about what good EoL care is, is generally viewed as problematic and the increasing number of articles that have documented this diversity call for standardisation. But this can lead to ignoring different ideas and practices and to promoting one specific version of EoL care which increases the risk that it will be used for ideological ends. This was the concern of the respondents who expressed doubts about the benefit of defining EoL care. Their reservation to defining EoL care was about fixing reality, whereby it gets removed from its context and loses all creativity and sensitivity to the particularities of which care consists.

### Limitations

The findings of this paper are based on a survey that used a qualitative approach. It contained open questions and respondents had the opportunity to make their answers as elaborate as they found necessary. Some respondents provided explanations that approached the length and depth of essays. This gave evidence of enthusiasm to contribute to this field, and added greatly to the value of the insights gained. However, due to this qualitative approach, only certain issues could be analysed quantitatively. The purposive and snowball strategy was also both a strength and a limitation of the study. Although we targeted a European expert group, this strategy had the effect that it also reached people outside Europe. We included their views in the analysis as the focus was on cultural diversity rather than European identity. However these do not represent diversity of global views but only those that chose to respond to a survey with an essentially European recruitment strategy. Due to the varying response rate of different countries and the number of respondents gathered through snowball recruitment, this is not a representative sample.

### Conclusions

The analysis of the findings on the definitions that are in use in a variety of cultural contexts confirmed earlier studies that there is no consensus on the terms for EoL care nor on the components of its definition. Earlier analyses of definitions related this to the changes in meanings the concept underwent through time. Our analysis has shown that the geographical spread of EoL care, across Europe and more globally, contributes to the diversity in how EoL care is understood. The analysis led to the identification of elements that are problematic in the definition of EoL care such as the specification of time frames or the boundaries between cure and care. This argues for an integrated approach to EoL care where prevention, cure, and care coexist. Such an approach is capable of encompassing all life-threatening illness. But EoL care is a universal concern and needs to be inclusive of a diversity of views from people with different cultural backgrounds.

Defining EoL care is essentially an ethical undertaking. Currently, EoL care is lacking the evidence and language to support a definition which embraces the various practices that have developed under its name. Research into the diversity of perspectives and practices of EoL care can help to develop a shared language to capture its specificity and its ethical basis.

## Supporting Information

Table S1 **Number of responses and response rate by mode of contact.**
(DOCX)

Table S2 **Number of responses by country.**
(DOCX)

Table S3 **Length of responses to the question on definitions.**
(DOCX)

Table S4 **Number of responses by self-identified professional category.**
(DOCX)
