# Supplementary material for: Diversity in Defining End of Life Care: An Obstacle or the Way Forward?
Source: PLoS One. 2013 Jul 3;8(7):e68002. doi: 10.1371/journal.pone.0068002 (PMC3700860; doi:10.1371/journal.pone.0068002)
Supplement: Table S1 — Number of responses and response rate by mode of contact. (DOCX) [file pone.0068002.s001.docx]

Table S1. Number of responses and response rate by mode of contact

| **Mode of contact** | **Number contacted** | **Number of responses** | **Response rate** | **Percentage of total responses** |
| --- | --- | --- | --- | --- |
| Snowball | 234 | 81 | 35.04 | 48.81 |
| Publication | 195 | 54 | 27.69 | 32.14 |
| Conference | 25 | 9 | 36.00 | 5.36 |
| Call for expertise | 57 | 23 | 40.35 | 13.69 |
| **Total** | **511** | **167** | **32.88** | **100** |
