# Supplementary material for: Diversity in Defining End of Life Care: An Obstacle or the Way Forward?
Source: PLoS One. 2013 Jul 3;8(7):e68002. doi: 10.1371/journal.pone.0068002 (PMC3700860; doi:10.1371/journal.pone.0068002)
Supplement: Table S2 — Number of responses by country. (DOCX) [file pone.0068002.s002.docx]

Table S2. Number of responses by country

| **Country** | **Number contacted** | **Number of responses** | **Percentage of total responses** |
| --- | --- | --- | --- |
| Argentina | 1 | 1 | 0.60 |
| Austria | 8 | 1 | 0.60 |
| Belgium | 62 | 14 | 8.33 |
| Canada | 3 | 1 | 0.60 |
| Chile | 1 | 0 | 0.00 |
| China | 4 | 2 | 1.19 |
| Denmark | 3 | 1 | 0.60 |
| France | 5 | 1 | 0.60 |
| Germany | 64 | 17 | 10.12 |
| Greece | 1 | 0 | 0.00 |
| India | 6 | 4 | 2.38 |
| Ireland | 7 | 6 | 3.57 |
| Israel | 6 | 2 | 1.19 |
| Italy | 44 | 16 | 9.52 |
| Luxemburg | 7 | 0 | 0.00 |
| Moldova | 1 | 0 | 0.00 |
| Netherlands | 49 | 20 | 11.90 |
| Norway | 55 | 9 | 5.36 |
| Peru | 1 | 0 | 0.00 |
| Portugal | 10 | 4 | 2.38 |
| South Africa | 1 | 0 | 0.00 |
| Spain | 65 | 21 | 12.50 |
| Sweden | 11 | 4 | 2.38 |
| Switzerland | 2 | 0 | 0.00 |
| Turkey | 5 | 1 | 0.60 |
| Uganda | 21 | 9 | 5.36 |
| UK | 68 | 33 | 20.24 |
| **Total** | **511** | **167** | **100** |
