# Supplementary material for: Diversity in Defining End of Life Care: An Obstacle or the Way Forward?
Source: PLoS One. 2013 Jul 3;8(7):e68002. doi: 10.1371/journal.pone.0068002 (PMC3700860; doi:10.1371/journal.pone.0068002)
Supplement: Table S3 — Length of responses to the question on definitions. (DOCX) [file pone.0068002.s003.docx]

Table S3: Length of responses to the question on definitions

| Total lines | 1135 |
| --- | --- |
| Average lines | 7 |
| Mode lines | 2 |
| Median lines | 4 |
| Average responses per site | 8 |
| Median (1-38) | 1 |
| Mode | 1 |
