# Supplementary material for: Diversity in Defining End of Life Care: An Obstacle or the Way Forward?
Source: PLoS One. 2013 Jul 3;8(7):e68002. doi: 10.1371/journal.pone.0068002 (PMC3700860; doi:10.1371/journal.pone.0068002)
Supplement: Table S4 — Number of responses by self-identified professional category. (DOCX) [file pone.0068002.s004.docx]

Table S4. Number of responses by self-identified professional category

| **Profession** | **Number of responses** | **Percentage of total responses** |
| --- | --- | --- |
| Academic | 66 | 39.29 |
| Academic and clinical practitioner | 39 | 23.21 |
| Academic and non-clinical practitioner | 3 | 1.79 |
| Clinical practitioner | 29 | 17.26 |
| Non-clinical practitioner | 15 | 9.52 |
| Other | 15 | 8.93 |
| **Total** | **167** | **100** |
